# Poly[bis­(piperazine-1,4-diium) [(μ_4_-*cyclo*-hexa­phosphato)dilithium] tetra­hydrate]

**DOI:** 10.1107/S1600536813011756

**Published:** 2013-05-11

**Authors:** Iness Ameur, Sonia Abid, Salem S. Al-Deyab, Mohamed Rzaigui

**Affiliations:** a Laboratoire de Chimie des Matériaux, Faculté des Sciences de Bizerte, 7021 Zarzouna Bizerte, Tunisia; b Petrochemical Research Chair, College of Science, King Saud University, Riyadh, Saudi Arabia

## Abstract

In the title compound, {(C_4_H_12_N_2_)_2_[Li_2_(P_6_O_18_)]·4H_2_O}_*n*_, the phosphate ring anion, located around an inversion center, adopts a chair conformation. Adjacent P_6_O_18_ rings are linked *via* corner-sharing by LiO_4_ tetra­hedra, generating anionic porous {[Li_2_(P_6_O_18_)]^4−^}_*n*_ layers parallel to (101). The piperazine-1,4-diium cations occupy the pores and develop hydrogen bonds with the inorganic framework. An extensive network of N—H⋯O and O—H⋯O hydrogen-bonding inter­actions link the components into a three-dimensional network and additional stabilization is provided by weak C—H⋯O hydrogen bonds.

## Related literature

For applications of compounds with open-framework structures, see: Assani *et al.* (2012 ▶); Mahesh *et al.* (2002 ▶); Natarajan (2000 ▶). For related structures with cyclo­hexa­phosphate rings, see: Abid *et al.* (2011 ▶), Amri *et al.* (2009 ▶); Marouani *et al.* (2010 ▶); For related structures with piperazine rings, see: Essid *et al.* (2010 ▶), Xu *et al.* (2007 ▶). For the synthesis of the precursor, see: Schülke & Kayser (1985 ▶).
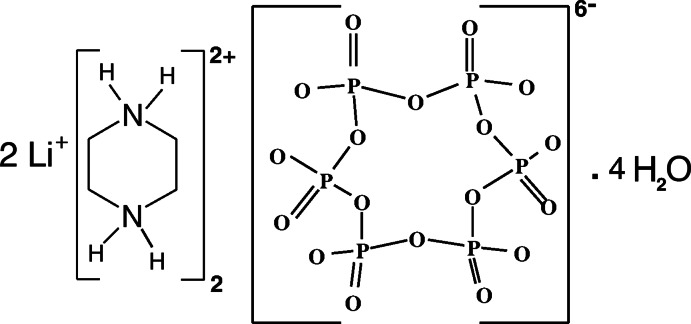


## Experimental

### 

#### Crystal data

(C_4_H_12_N_2_)_2_[Li_2_(P_6_O_18_)]·4H_2_O
*M* *_r_* = 736.08
Monoclinic, 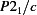

*a* = 10.245 (3) Å
*b* = 12.966 (4) Å
*c* = 10.910 (4) Å
β = 111.00 (3)°
*V* = 1352.8 (7) Å^3^
*Z* = 2
Ag *K*α radiation
λ = 0.56085 Å
μ = 0.26 mm^−1^
*T* = 293 K
0.50 × 0.40 × 0.30 mm

#### Data collection

Enraf–Nonius CAD-4 diffractometer
7884 measured reflections
6469 independent reflections
3904 reflections with *I* > 2σ(*I*)
*R*_int_ = 0.038
2 standard reflections every 120 min intensity decay: 1%

#### Refinement

*R*[*F* ^2^ > 2σ(*F* ^2^)] = 0.050
*wR*(*F* ^2^) = 0.128
*S* = 1.00
6469 reflections
202 parameters
6 restraints
H atoms treated by a mixture of independent and constrained refinement
Δρ_max_ = 0.57 e Å^−3^
Δρ_min_ = −0.47 e Å^−3^

### 

Data collection: *CAD-4 EXPRESS* (Enraf–Nonius, 1994 ▶); cell refinement: *CAD-4 EXPRESS*; data reduction: *XCAD4* (Harms & Wocadlo, 1995 ▶); program(s) used to solve structure: *SHELXS86* (Sheldrick, 2008 ▶); program(s) used to refine structure: *SHELXL97* (Sheldrick, 2008 ▶); molecular graphics: *ORTEP-3 for Windows* (Farrugia, 2012 ▶); software used to prepare material for publication: *WinGX* (Farrugia, 2012 ▶).

## Supplementary Material

Crystal structure: contains datablock(s) I, global. DOI: 10.1107/S1600536813011756/pk2476sup1.cif

Structure factors: contains datablock(s) I. DOI: 10.1107/S1600536813011756/pk2476Isup2.hkl

Additional supplementary materials:  crystallographic information; 3D view; checkCIF report

## Figures and Tables

**Table 1:** Hydrogen-bond geometry (Å, °)

*D*—H⋯*A*	*D*—H	H⋯*A*	*D*⋯*A*	*D*—H⋯*A*
N1—H2*A*⋯O8	0.90	1.99	2.792 (3)	147
N1—H2*B*⋯O7^i^	0.90	1.88	2.763 (3)	166
N2—H3*A*⋯O11	0.90	1.83	2.707 (4)	165
N2—H3*B*⋯O4^ii^	0.90	1.91	2.802 (2)	168
O10—H110⋯O1^iii^	0.84 (4)	1.99 (4)	2.792 (4)	159 (5)
O11—H111⋯O5^iii^	0.84 (3)	2.49 (4)	3.001 (3)	120 (3)
O11—H111⋯O8^iii^	0.84 (3)	2.07 (3)	2.826 (3)	150 (4)
O10—H210⋯O5^i^	0.82 (4)	1.95 (4)	2.764 (3)	170 (4)
O11—H211⋯O10^iii^	0.86 (4)	1.87 (4)	2.720 (4)	170 (4)
C1—H1*A*⋯O6^iv^	0.97	2.51	3.273 (4)	135
C4—H1*D*⋯O10	0.97	2.56	3.227 (4)	126
C2—H2*C*⋯O2^v^	0.97	2.29	3.078 (3)	137
C3—H4*B*⋯O5^iii^	0.97	2.46	3.324 (4)	148

Symmetry codes: (i) 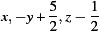; (ii) 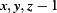; (iii) 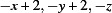; (iv) 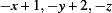; (v) 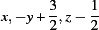.

**Fig. 1.**
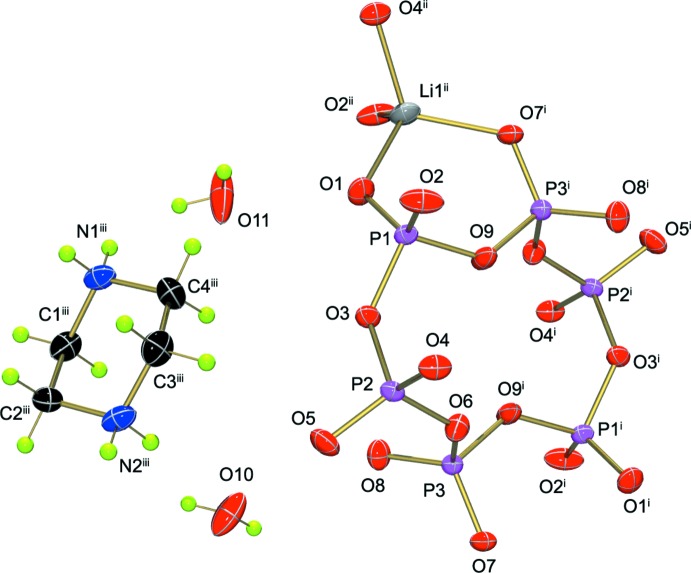
An ellipsoid plot of (I) with displacement ellipsoids for non-H atoms drawn at the 30% probability level.

**Fig. 2.**
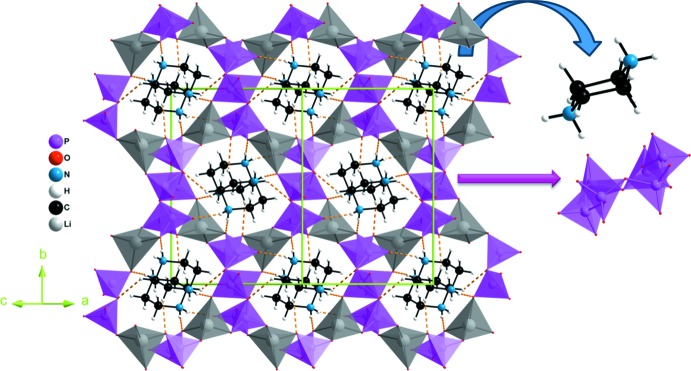
Projection in the [101] direction, showing the pores and their occupancy by the organic groups.

**Fig. 3.**
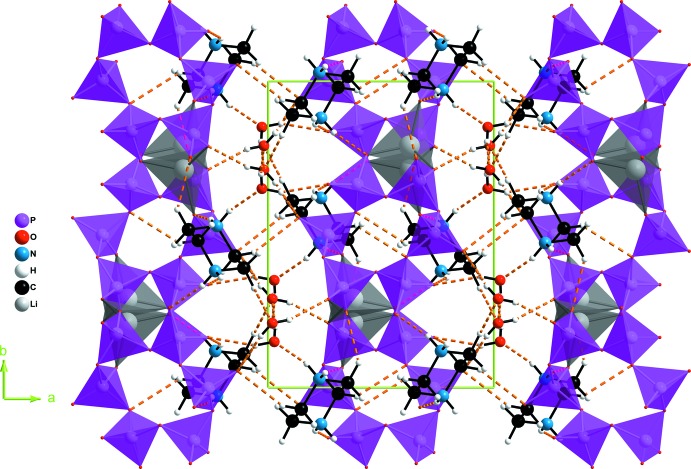
Projection of the framework of (I) along the c direction.

## supplementary crystallographic information

### Comment

The area of framework materials continues to be of interest not only because of the wide variety of structures but also due to their potential applications in the areas of catalysis, sorption and separation processes (Mahesh *et al.*, 2002) Natarajan, 2000). Much attention has been devoted to the synthesis of open-framework phosphates which exhibit a rich structural diversity and have been widely studied as catalysts, ion-exchangers and as positive electrode in the lithium and sodium batteries (Assani *et al.*, 2012). Within this family of compounds, the resulting anionic frameworks, generally constructed from PO_4_ tetrahedra that are vertex linked with MO_n_ polyhedra (with n = 4, 5 and 6), generate pores and channels offering suitable environment to accommodate different other cations. The piperazine (C_4_N_2_H_10_), which is a common heterocyclic nitrogen compound, has been indicated as excellent template for preparing microporous materials (Xu *et al.*, 2007). The crystal structure reported here gives another illustration of this type of material. The corresponding compound, (C_4_H_12_N_2_)_2_Li_2_P_6_O_18_.4H_2_O (I), is an organic-inorganic hybrid built of two main cyclic components, C_4_H_12_N_2_ and P_6_O_18_ (Fig. 1). The phosphoric rings are interconnected by the Li^+^ cations *via* LiO_4_ tetrahedra sharing corners to form a two-dimensional inorganic framework extending along the (101) plane as shown in Fig. 2. The diprotonated (C_4_H_12_N_2_)^2+^ cations are trapped within the 10-membered ring pore of the layer, whereas the water molecules are located in the interlayer region and are grafted onto the framework oxygen atoms through hydrogen bonds (Fig. 3). The asymmetric unit of this atomic arrangement is built of one half of the P_6_O_18_ ring lying on an inversion center (1/2, 1/2, 1/2), one Li^+^ cation, two water molecules and one piperazine-1,4-diium cation. The organic and inorganic rings adopt a chair conformation with different geometrical characteristics due to their different size and flexibility. However, the P_6_O_18_ ring has (P–O and O–O) distances and (O–P–O, P–O–P and P–P–P angles) comparable to those observed in other cyclohexaphosphates having the same internal inversion symmetry (Abid *et al.*, 2011; Amri *et al.*, 2009; Marouani *et al.*, 2010). The LiO_4_ tetrahedra is slightly distorted with Li–O distances ranging from 1.877 (4) to 1.969 (4) Å. The smallest distance between two tetrahedral centers is 5.548 (2) Å. The organic ring has for carbon atoms (C1, C2, C3 and C4) almost coplanar (r.m.s. deviation from the mean plane = 0.014 Å) and N1 and N2 displaced from the plane by 0.672 (2) and -0.663 (2) Å, respectively. These characteristics do not differ from those particular values observed in other compounds of the piperazinium despite the different constraints of their solid states (Essid *et al.*, 2010).

### Experimental

Crystals of the title compound were prepared by adding dropwise and stirring an ethanolic solution (5 mL) of piperazine (10 mmol) then an aquous solution (10 mL) of KOH (10 mmol) to an aqueous solution (10 mL) of cyclohexaphosphoric acid (5 mmol). Colourless prismatic crystals were obtained after a slow evaporation over a few days at ambient temperature. The cyclohexaphosphoric acid H_6_P_6_O_18_,was produced from Li_6_P_6_O_18_.6H_2_O, prepared according to the procedure of Schülke and Kayser (Schülke & Kayser, 1985), through an ion-exchange resin in H-state (Amberlite IR 120).

### Refinement

N and C-bound H atoms were positioned geometrically (N–H = 0.90 Å, C–H = 0.97 Å) and allowed to ride on their parent atoms, with *U*_iso_(H) = 1.2 *U*_eq_(C,*N*). The bond distances of O–H and distance between two H atoms from each water molecules was restrained to be 0.85 and 1.37 Å with the default deviation respectively and with *U*_iso_(H) = 1.5 *U*_eq_ (O).

### Crystal data

**Table:**

(C_4_H_12_N_2_)_2_[Li_2_(P_6_O_18_)]·4H_2_O	*F*(000) = 760
*M**_r_* = 736.08	*D*_x_ = 1.807 Mg m^−^^3^
Monoclinic, *P*2_1_/*c*	Ag *K*α radiation, λ = 0.56085 Å
Hall symbol: -P 2ybc	Cell parameters from 25 reflections
*a* = 10.245 (3) Å	θ = 9.1–10.9°
*b* = 12.966 (4) Å	µ = 0.26 mm^−^^1^
*c* = 10.910 (4) Å	*T* = 293 K
β = 111.00 (3)°	Parallelepiped, colourless
*V* = 1352.8 (7) Å^3^	0.50 × 0.40 × 0.30 mm
*Z* = 2	

### Data collection

**Table:**

Enraf–Nonius CAD-4 diffractometer	*R*_int_ = 0.038
Radiation source: fine-focus sealed tube	θ_max_ = 28.0°, θ_min_ = 2.0°
Graphite monochromator	*h* = −17→15
non–profiled ω–scans	*k* = −3→21
7884 measured reflections	*l* = 0→18
6469 independent reflections	2 standard reflections every 120 min
3904 reflections with *I* > 2σ(*I*)	intensity decay: 1%

### Refinement

**Table:**

Refinement on *F*^2^	Primary atom site location: structure-invariant direct methods
Least-squares matrix: full	Secondary atom site location: difference Fourier map
*R*[*F*^2^ > 2σ(*F*^2^)] = 0.050	Hydrogen site location: inferred from neighbouring sites
*wR*(*F*^2^) = 0.128	H atoms treated by a mixture of independent and constrained refinement
*S* = 1.00	*w* = 1/[σ^2^(*F*_o_^2^) + (0.059*P*)^2^] where *P* = (*F*_o_^2^ + 2*F*_c_^2^)/3
6469 reflections	(Δ/σ)_max_ = 0.001
202 parameters	Δρ_max_ = 0.57 e Å^−^^3^
6 restraints	Δρ_min_ = −0.47 e Å^−^^3^

### Special details

**Table:**

Geometry. All e.s.d.'s (except the e.s.d. in the dihedral angle between two l.s. planes) are estimated using the full covariance matrix. The cell e.s.d.'s are taken into account individually in the estimation of e.s.d.'s in distances, angles and torsion angles; correlations between e.s.d.'s in cell parameters are only used when they are defined by crystal symmetry. An approximate (isotropic) treatment of cell e.s.d.'s is used for estimating e.s.d.'s involving l.s. planes.
Refinement. Refinement of *F*^2^ against ALL reflections. The weighted *R*-factor *wR* and goodness of fit *S* are based on *F*^2^, conventional *R*-factors *R* are based on *F*, with *F* set to zero for negative *F*^2^. The threshold expression of *F*^2^ > σ(*F*^2^) is used only for calculating *R*-factors(gt) *etc*. and is not relevant to the choice of reflections for refinement. *R*-factors based on *F*^2^ are statistically about twice as large as those based on *F*, and *R*- factors based on ALL data will be even larger.

### Fractional atomic coordinates and isotropic or equivalent isotropic displacement parameters (Å^2^)

**Table:**

	*x*	*y*	*z*	*U*_iso_*/*U*_eq_	
P1	0.67085 (5)	0.82511 (4)	0.10243 (4)	0.01597 (10)	
P2	0.73450 (5)	0.99726 (4)	0.28486 (5)	0.01656 (10)	
P3	0.60626 (5)	1.16305 (4)	0.09894 (5)	0.01660 (10)	
O3	0.75517 (14)	0.92532 (11)	0.17300 (12)	0.0195 (3)	
O6	0.61176 (15)	1.07330 (12)	0.20122 (14)	0.0232 (3)	
O5	0.86457 (16)	1.05454 (13)	0.34765 (15)	0.0287 (3)	
O2	0.6560 (2)	0.75209 (12)	0.19975 (15)	0.0325 (4)	
O4	0.67240 (16)	0.93559 (11)	0.36583 (13)	0.0227 (3)	
O1	0.73374 (16)	0.78864 (13)	0.00746 (14)	0.0267 (3)	
O8	0.73427 (15)	1.16386 (13)	0.06704 (14)	0.0251 (3)	
O7	0.56459 (15)	1.26004 (11)	0.14913 (14)	0.0232 (3)	
O9	0.52184 (15)	0.87728 (12)	0.02453 (14)	0.0265 (3)	
N1	0.7657 (2)	1.11939 (14)	−0.17128 (17)	0.0267 (4)	
H2A	0.7908	1.1407	−0.0875	0.032*	
H2B	0.7086	1.1673	−0.2230	0.032*	
C1	0.6902 (3)	1.01996 (18)	−0.1870 (2)	0.0304 (5)	
H1A	0.6079	1.0284	−0.1637	0.036*	
H1B	0.7501	0.9687	−0.1291	0.036*	
C4	0.8925 (2)	1.1093 (2)	−0.2064 (2)	0.0354 (5)	
H1C	0.9576	1.0617	−0.1464	0.042*	
H1D	0.9381	1.1758	−0.1984	0.042*	
C3	0.8530 (3)	1.0702 (2)	−0.3449 (2)	0.0335 (5)	
H4A	0.7966	1.1217	−0.4054	0.040*	
H4B	0.9370	1.0592	−0.3647	0.040*	
N2	0.7735 (2)	0.97230 (15)	−0.36309 (18)	0.0290 (4)	
H3A	0.8288	0.9229	−0.3127	0.035*	
H3B	0.7476	0.9522	−0.4474	0.035*	
Li1	0.6251 (4)	0.7890 (3)	0.3536 (3)	0.0246 (7)	
C2	0.6474 (3)	0.98435 (18)	−0.3276 (2)	0.0307 (5)	
H2C	0.5984	0.9190	−0.3383	0.037*	
H2D	0.5846	1.0344	−0.3853	0.037*	
O10	1.0185 (2)	1.28956 (19)	0.0074 (2)	0.0502 (6)	
O11	0.9729 (2)	0.8504 (2)	−0.1959 (3)	0.0704 (9)	
H110	1.094 (3)	1.281 (4)	−0.006 (4)	0.106*	
H210	0.967 (4)	1.330 (3)	−0.047 (4)	0.106*	
H111	1.058 (2)	0.863 (4)	−0.179 (4)	0.106*	
H211	0.969 (5)	0.802 (3)	−0.144 (4)	0.106*	

### Atomic displacement parameters (Å^2^)

**Table:**

	*U*^11^	*U*^22^	*U*^33^	*U*^12^	*U*^13^	*U*^23^
P1	0.0187 (2)	0.0152 (2)	0.01382 (18)	0.00116 (17)	0.00556 (15)	−0.00189 (16)
P2	0.0190 (2)	0.0153 (2)	0.01505 (19)	−0.00225 (17)	0.00565 (15)	−0.00241 (16)
P3	0.0162 (2)	0.0158 (2)	0.01738 (19)	0.00004 (17)	0.00551 (15)	−0.00147 (16)
O3	0.0212 (6)	0.0209 (6)	0.0181 (6)	−0.0030 (5)	0.0091 (5)	−0.0058 (5)
O6	0.0217 (7)	0.0207 (7)	0.0302 (7)	0.0029 (5)	0.0128 (6)	0.0062 (6)
O5	0.0250 (7)	0.0285 (8)	0.0271 (7)	−0.0091 (6)	0.0027 (6)	−0.0082 (6)
O2	0.0594 (12)	0.0183 (7)	0.0234 (7)	−0.0060 (7)	0.0191 (7)	−0.0004 (6)
O4	0.0337 (8)	0.0192 (6)	0.0185 (6)	−0.0016 (6)	0.0137 (6)	0.0005 (5)
O1	0.0254 (7)	0.0347 (8)	0.0224 (6)	0.0018 (6)	0.0114 (6)	−0.0110 (6)
O8	0.0205 (6)	0.0345 (8)	0.0224 (6)	−0.0001 (6)	0.0103 (5)	0.0014 (6)
O7	0.0242 (7)	0.0161 (6)	0.0275 (7)	0.0000 (5)	0.0071 (6)	−0.0063 (5)
O9	0.0196 (7)	0.0258 (7)	0.0267 (7)	0.0058 (6)	−0.0007 (5)	−0.0122 (6)
N1	0.0352 (10)	0.0237 (9)	0.0192 (7)	0.0056 (7)	0.0076 (7)	−0.0028 (6)
C1	0.0372 (12)	0.0262 (10)	0.0362 (11)	0.0027 (9)	0.0235 (10)	0.0068 (9)
C4	0.0259 (11)	0.0373 (13)	0.0395 (12)	−0.0065 (10)	0.0075 (9)	−0.0058 (10)
C3	0.0349 (12)	0.0400 (13)	0.0333 (11)	0.0017 (10)	0.0215 (10)	0.0040 (10)
N2	0.0398 (11)	0.0255 (9)	0.0213 (8)	0.0097 (8)	0.0104 (7)	−0.0012 (7)
Li1	0.0339 (19)	0.0211 (17)	0.0196 (15)	−0.0037 (15)	0.0106 (14)	0.0038 (13)
C2	0.0304 (11)	0.0208 (10)	0.0384 (12)	−0.0015 (8)	0.0091 (9)	−0.0031 (9)
O10	0.0547 (13)	0.0556 (13)	0.0526 (12)	0.0314 (11)	0.0343 (11)	0.0273 (10)
O11	0.0229 (9)	0.0848 (19)	0.1003 (19)	0.0123 (11)	0.0183 (11)	0.0638 (16)

### Geometric parameters (Å, º)

**Table:**

P1—O2	1.4707 (16)	N1—H2B	0.9000
P1—O1	1.4801 (15)	C1—C2	1.509 (3)
P1—O3	1.5970 (15)	C1—H1A	0.9700
P1—O9	1.6063 (16)	C1—H1B	0.9700
P1—Li1	2.978 (4)	C4—C3	1.505 (3)
P1—Li1^i^	2.978 (4)	C4—H1C	0.9700
P2—O5	1.4636 (16)	C4—H1D	0.9700
P2—O4	1.4925 (15)	C3—N2	1.483 (3)
P2—O6	1.6011 (16)	C3—H4A	0.9700
P2—O3	1.6091 (14)	C3—H4B	0.9700
P2—Li1	3.117 (4)	N2—C2	1.483 (3)
P3—O8	1.4725 (15)	N2—H3A	0.9000
P3—O7	1.4937 (15)	N2—H3B	0.9000
P3—O9^ii^	1.5945 (16)	Li1—O1^iv^	1.931 (4)
P3—O6	1.5991 (15)	Li1—O7^v^	1.969 (4)
P3—Li1^iii^	3.071 (4)	Li1—P1^iv^	2.978 (4)
O2—Li1	1.877 (4)	Li1—P3^v^	3.071 (4)
O4—Li1	1.954 (4)	C2—H2C	0.9700
O1—Li1^i^	1.931 (4)	C2—H2D	0.9700
O7—Li1^iii^	1.969 (4)	O10—H110	0.846 (18)
O9—P3^ii^	1.5945 (16)	O10—H210	0.822 (18)
N1—C1	1.482 (3)	O11—H111	0.840 (18)
N1—C4	1.485 (3)	O11—H211	0.851 (18)
N1—H2A	0.9000		
			
O2—P1—O1	118.91 (10)	N1—C4—H1C	109.6
O2—P1—O3	110.72 (9)	C3—C4—H1C	109.6
O1—P1—O3	107.54 (9)	N1—C4—H1D	109.6
O2—P1—O9	109.16 (11)	C3—C4—H1D	109.6
O1—P1—O9	109.50 (9)	H1C—C4—H1D	108.2
O3—P1—O9	99.19 (8)	N2—C3—C4	111.03 (19)
O1—P1—Li1	147.71 (11)	N2—C3—H4A	109.4
O3—P1—Li1	85.45 (9)	C4—C3—H4A	109.4
O9—P1—Li1	96.89 (10)	N2—C3—H4B	109.4
O2—P1—Li1^i^	108.38 (10)	C4—C3—H4B	109.4
O1—P1—Li1^i^	33.78 (10)	H4A—C3—H4B	108.0
O3—P1—Li1^i^	136.45 (9)	C3—N2—C2	111.29 (17)
O9—P1—Li1^i^	85.28 (10)	C3—N2—H3A	109.4
Li1—P1—Li1^i^	137.36 (7)	C2—N2—H3A	109.4
O5—P2—O4	120.31 (9)	C3—N2—H3B	109.4
O5—P2—O6	110.58 (10)	C2—N2—H3B	109.4
O4—P2—O6	104.65 (8)	H3A—N2—H3B	108.0
O5—P2—O3	107.69 (9)	O2—Li1—O1^iv^	114.5 (2)
O4—P2—O3	109.77 (9)	O2—Li1—O4	101.07 (17)
O6—P2—O3	102.40 (8)	O1^iv^—Li1—O4	113.3 (2)
O5—P2—Li1	132.28 (10)	O2—Li1—O7^v^	114.8 (2)
O4—P2—Li1	29.20 (9)	O1^iv^—Li1—O7^v^	99.89 (17)
O6—P2—Li1	113.21 (9)	O4—Li1—O7^v^	113.9 (2)
O3—P2—Li1	80.59 (9)	O2—Li1—P1	23.82 (7)
O8—P3—O7	118.45 (10)	O1^iv^—Li1—P1	130.56 (19)
O8—P3—O9^ii^	109.62 (9)	O4—Li1—P1	78.06 (12)
O7—P3—O9^ii^	109.20 (8)	O7^v^—Li1—P1	119.75 (16)
O8—P3—O6	111.01 (9)	O2—Li1—P1^iv^	131.81 (19)
O7—P3—O6	107.46 (9)	O1^iv^—Li1—P1^iv^	25.22 (7)
O9^ii^—P3—O6	99.40 (9)	O4—Li1—P1^iv^	117.81 (16)
O8—P3—Li1^iii^	147.42 (10)	O7^v^—Li1—P1^iv^	75.57 (12)
O7—P3—Li1^iii^	31.94 (9)	P1—Li1—P1^iv^	153.12 (15)
O9^ii^—P3—Li1^iii^	82.27 (9)	O2—Li1—P3^v^	113.75 (17)
O6—P3—Li1^iii^	96.05 (9)	O1^iv^—Li1—P3^v^	79.37 (12)
P1—O3—P2	129.92 (9)	O4—Li1—P3^v^	133.85 (18)
P3—O6—P2	132.05 (9)	O7^v^—Li1—P3^v^	23.66 (7)
P1—O2—Li1	125.14 (15)	P1—Li1—P3^v^	128.64 (13)
P2—O4—Li1	128.93 (14)	P1^iv^—Li1—P3^v^	57.37 (7)
P1—O1—Li1^i^	121.01 (15)	O2—Li1—P2	79.37 (12)
P3—O7—Li1^iii^	124.41 (14)	O1^iv^—Li1—P2	121.05 (17)
P3^ii^—O9—P1	130.33 (10)	O4—Li1—P2	21.87 (6)
C1—N1—C4	111.21 (18)	O7^v^—Li1—P2	126.83 (17)
C1—N1—H2A	109.4	P1—Li1—P2	56.88 (6)
C4—N1—H2A	109.4	P1^iv^—Li1—P2	134.19 (13)
C1—N1—H2B	109.4	P3^v^—Li1—P2	150.11 (14)
C4—N1—H2B	109.4	N2—C2—C1	109.55 (19)
H2A—N1—H2B	108.0	N2—C2—H2C	109.8
N1—C1—C2	109.47 (17)	C1—C2—H2C	109.8
N1—C1—H1A	109.8	N2—C2—H2D	109.8
C2—C1—H1A	109.8	C1—C2—H2D	109.8
N1—C1—H1B	109.8	H2C—C2—H2D	108.2
C2—C1—H1B	109.8	H110—O10—H210	111 (3)
H1A—C1—H1B	108.2	H111—O11—H211	107 (3)
N1—C4—C3	110.11 (19)		
			
O2—P1—O3—P2	−48.29 (15)	O3—P1—Li1—O1^iv^	−91.0 (2)
O1—P1—O3—P2	−179.72 (12)	O9—P1—Li1—O1^iv^	170.3 (2)
O9—P1—O3—P2	66.34 (13)	Li1^i^—P1—Li1—O1^iv^	79.8 (4)
Li1—P1—O3—P2	−29.92 (13)	O2—P1—Li1—O4	164.8 (3)
Li1^i^—P1—O3—P2	159.16 (13)	O1—P1—Li1—O4	135.73 (16)
O5—P2—O3—P1	160.31 (12)	O3—P1—Li1—O4	19.62 (12)
O4—P2—O3—P1	27.66 (14)	O9—P1—Li1—O4	−79.12 (13)
O6—P2—O3—P1	−83.09 (13)	Li1^i^—P1—Li1—O4	−169.63 (10)
Li1—P2—O3—P1	28.79 (13)	O2—P1—Li1—O7^v^	−84.4 (2)
O8—P3—O6—P2	8.74 (17)	O1—P1—Li1—O7^v^	−113.4 (2)
O7—P3—O6—P2	−122.24 (13)	O3—P1—Li1—O7^v^	130.49 (19)
O9^ii^—P3—O6—P2	124.08 (14)	O9—P1—Li1—O7^v^	31.8 (2)
Li1^iii^—P3—O6—P2	−152.78 (14)	Li1^i^—P1—Li1—O7^v^	−58.8 (3)
O5—P2—O6—P3	46.37 (16)	O2—P1—Li1—P1^iv^	35.2 (3)
O4—P2—O6—P3	177.31 (12)	O1—P1—Li1—P1^iv^	6.1 (4)
O3—P2—O6—P3	−68.15 (15)	O3—P1—Li1—P1^iv^	−110.0 (3)
Li1—P2—O6—P3	−153.10 (13)	O9—P1—Li1—P1^iv^	151.3 (3)
O1—P1—O2—Li1	162.77 (19)	Li1^i^—P1—Li1—P1^iv^	60.8 (5)
O3—P1—O2—Li1	37.5 (2)	O2—P1—Li1—P3^v^	−57.7 (2)
O9—P1—O2—Li1	−70.7 (2)	O1—P1—Li1—P3^v^	−86.8 (2)
Li1^i^—P1—O2—Li1	−162.0 (2)	O3—P1—Li1—P3^v^	157.13 (17)
O5—P2—O4—Li1	−123.45 (19)	O9—P1—Li1—P3^v^	58.39 (17)
O6—P2—O4—Li1	111.54 (19)	Li1^i^—P1—Li1—P3^v^	−32.1 (3)
O3—P2—O4—Li1	2.3 (2)	O2—P1—Li1—P2	158.8 (2)
O2—P1—O1—Li1^i^	79.75 (19)	O1—P1—Li1—P2	129.75 (16)
O3—P1—O1—Li1^i^	−153.48 (16)	O3—P1—Li1—P2	13.64 (6)
O9—P1—O1—Li1^i^	−46.64 (19)	O9—P1—Li1—P2	−85.09 (7)
Li1—P1—O1—Li1^i^	96.36 (17)	Li1^i^—P1—Li1—P2	−175.60 (15)
O8—P3—O7—Li1^iii^	160.49 (16)	O5—P2—Li1—O2	−110.53 (15)
O9^ii^—P3—O7—Li1^iii^	34.12 (19)	O4—P2—Li1—O2	172.7 (3)
O6—P3—O7—Li1^iii^	−72.80 (18)	O6—P2—Li1—O2	94.41 (14)
O2—P1—O9—P3^ii^	−83.58 (15)	O3—P2—Li1—O2	−5.13 (12)
O1—P1—O9—P3^ii^	48.19 (17)	O5—P2—Li1—O1^iv^	1.8 (3)
O3—P1—O9—P3^ii^	160.59 (13)	O4—P2—Li1—O1^iv^	−75.0 (2)
Li1—P1—O9—P3^ii^	−112.92 (15)	O6—P2—Li1—O1^iv^	−153.24 (17)
Li1^i^—P1—O9—P3^ii^	24.26 (15)	O3—P2—Li1—O1^iv^	107.2 (2)
C4—N1—C1—C2	−59.4 (2)	O5—P2—Li1—O4	76.8 (2)
C1—N1—C4—C3	57.2 (3)	O6—P2—Li1—O4	−78.3 (2)
N1—C4—C3—N2	−55.0 (3)	O3—P2—Li1—O4	−177.8 (2)
C4—C3—N2—C2	56.3 (3)	O5—P2—Li1—O7^v^	136.35 (18)
P1—O2—Li1—O1^iv^	−137.38 (19)	O4—P2—Li1—O7^v^	59.6 (2)
P1—O2—Li1—O4	−15.2 (3)	O6—P2—Li1—O7^v^	−18.7 (2)
P1—O2—Li1—O7^v^	107.9 (2)	O3—P2—Li1—O7^v^	−118.2 (2)
P1—O2—Li1—P1^iv^	−159.56 (17)	O5—P2—Li1—P1	−119.08 (11)
P1—O2—Li1—P3^v^	133.82 (15)	O4—P2—Li1—P1	164.1 (2)
P1—O2—Li1—P2	−17.96 (18)	O6—P2—Li1—P1	85.86 (9)
P2—O4—Li1—O2	−7.3 (3)	O3—P2—Li1—P1	−13.68 (6)
P2—O4—Li1—O1^iv^	115.71 (19)	O5—P2—Li1—P1^iv^	29.2 (3)
P2—O4—Li1—O7^v^	−130.98 (17)	O4—P2—Li1—P1^iv^	−47.53 (18)
P2—O4—Li1—P1	−13.53 (18)	O6—P2—Li1—P1^iv^	−125.81 (18)
P2—O4—Li1—P1^iv^	143.28 (13)	O3—P2—Li1—P1^iv^	134.6 (2)
P2—O4—Li1—P3^v^	−146.51 (15)	O5—P2—Li1—P3^v^	129.8 (2)
O1—P1—Li1—O2	−29.0 (3)	O4—P2—Li1—P3^v^	53.0 (2)
O3—P1—Li1—O2	−145.1 (2)	O6—P2—Li1—P3^v^	−25.3 (3)
O9—P1—Li1—O2	116.1 (2)	O3—P2—Li1—P3^v^	−124.8 (3)
Li1^i^—P1—Li1—O2	25.6 (3)	C3—N2—C2—C1	−58.0 (2)
O2—P1—Li1—O1^iv^	54.2 (2)	N1—C1—C2—N2	59.0 (2)
O1—P1—Li1—O1^iv^	25.1 (4)		

Symmetry codes: (i) *x*, −*y*+3/2, *z*−1/2; (ii) −*x*+1, −*y*+2, −*z*; (iii) −*x*+1, *y*+1/2, −*z*+1/2; (iv) *x*, −*y*+3/2, *z*+1/2; (v) −*x*+1, *y*−1/2, −*z*+1/2.

### Hydrogen-bond geometry (Å, º)

**Table:**

*D*—H···*A*	*D*—H	H···*A*	*D*···*A*	*D*—H···*A*
N1—H2*A*···O8	0.90	1.99	2.792 (3)	147
N1—H2*B*···O7^vi^	0.90	1.88	2.763 (3)	166
N2—H3*A*···O11	0.90	1.83	2.707 (4)	165
N2—H3*B*···O4^vii^	0.90	1.91	2.802 (2)	168
O10—H110···O1^viii^	0.84 (4)	1.99 (4)	2.792 (4)	159 (5)
O11—H111···O5^viii^	0.84 (3)	2.49 (4)	3.001 (3)	120 (3)
O11—H111···O8^viii^	0.84 (3)	2.07 (3)	2.826 (3)	150 (4)
O10—H210···O5^vi^	0.82 (4)	1.95 (4)	2.764 (3)	170 (4)
O11—H211···O10^viii^	0.86 (4)	1.87 (4)	2.720 (4)	170 (4)
C1—H1*A*···O6^ii^	0.97	2.51	3.273 (4)	135
C4—H1*D*···O10	0.97	2.56	3.227 (4)	126
C2—H2*C*···O2^i^	0.97	2.29	3.078 (3)	137
C3—H4*B*···O5^viii^	0.97	2.46	3.324 (4)	148

Symmetry codes: (i) *x*, −*y*+3/2, *z*−1/2; (ii) −*x*+1, −*y*+2, −*z*; (vi) *x*, −*y*+5/2, *z*−1/2; (vii) *x*, *y*, *z*−1; (viii) −*x*+2, −*y*+2, −*z*.
